# The telomeric protein AKTIP interacts with A- and B-type lamins and is involved in regulation of cellular senescence

**DOI:** 10.1098/rsob.160103

**Published:** 2016-08-10

**Authors:** Romina Burla, Mariateresa Carcuro, Mattia La Torre, Federica Fratini, Marco Crescenzi, Maria Rosaria D'Apice, Paola Spitalieri, Grazia Daniela Raffa, Letizia Astrologo, Giovanna Lattanzi, Enrico Cundari, Domenico Raimondo, Annamaria Biroccio, Maurizio Gatti, Isabella Saggio

**Affiliations:** 1 Dipartimento di Biologia e Biotecnologie “C. Darwin”, Sapienza Università di Roma, 00185Italy; 2 Istituto Pasteur Fondazione Cenci Bolognetti, Rome 00185Italy; 3 Istituto di Biologia e Patologia Molecolari CNR Roma, 00185Italy; 4 Istituto Superiore di Sanità, Rome, 00185Italy; 5 Fondazione Policlinico Tor Vergata, Roma, 00133Italy; 6 Dipartimento di Biomedicina e Prevenzione, Università di Roma Tor Vergata, Roma 00133, Italy; 7 Istituto di Genetica Molecolare, CNR Bologna, 40136Italy; 8 Dipartimento di Medicina Molecolare, Sapienza, Università di Roma, Rome 00185Italy; 9 Unità di Oncogenomica ed Epigenetica, Istituto Nazionale Tumori Regina Elena, Roma 00144, Italy

**Keywords:** lamin, telomeres, laminopathies, progeria, nuclear lamina, cell senescence

## Abstract

AKTIP is a shelterin-interacting protein required for replication of telomeric DNA. Here, we show that AKTIP biochemically interacts with A- and B-type lamins and affects lamin A, but not lamin C or B, expression. In interphase cells, AKTIP localizes at the nuclear rim and in discrete regions of the nucleoplasm just like lamins. Double immunostaining revealed that AKTIP partially co-localizes with lamin B1 and lamin A/C in interphase cells, and that proper AKTIP localization requires functional lamin A. In mitotic cells, AKTIP is enriched at the spindle poles and at the midbody of late telophase cells similar to lamin B1. AKTIP-depleted cells show senescence-associated markers and recapitulate several aspects of the progeroid phenotype. Collectively, our results indicate that AKTIP is a new player in lamin-related processes, including those that govern nuclear architecture, telomere homeostasis and cellular senescence.

## 1. Background

The nuclear lamina is a structure located between the nuclear membrane and the chromatin; it plays a scaffolding role within the nucleus and has been implicated in diverse processes including the control of nuclear architecture, DNA replication and repair, transcriptional regulation, and maintenance of telomere homeostasis [1–6]. A- and B-type lamins are the main components of the nuclear lamina; B-type lamins are expressed in most cell types, whereas lamin A/C expression is restricted to differentiated cells [7,8]. In interphase cells, lamins are mainly detected at the nuclear periphery, but are also found in discrete intranuclear aggregates associated with DNA replication foci or transcriptionally active areas [7]. At the onset of mitosis the lamina disassembles; B-type lamins concentrate in the spindle matrix, whereas A-type lamins remain diffuse in the cytoplasm. At the end of mitosis (telophase), the lamins reassemble to form the nuclear lamina [7,9,10].

In humans, there are three lamin genes: *LMNA*, *LMNB1* and *LMNB2*. Lamins A and C are alternatively spliced isoforms of the *LMNA* gene. Lamins B1 and B2 are the products of the *LMNB1* and *LMNB2* genes. Lamins A, B1 and B2 are initially synthesized as precursors, known as prelamins, which are processed into mature lamins through a sequential series of post-translational modifications [1]. Mutations in the human lamin genes result in a wide range of diseases, including the Hutchinson-Gilford progeria syndrome (HGPS) [11] and mandibuloacral dysplasia type A (MADA) [12]. HGPS patients suffer from premature ageing; their cells exhibit an accumulation of a truncated form of lamin A, which causes early senescence, dismorphic nuclei, abnormal intranuclear chromatin distribution and telomere attrition [3,13]. MADA patients share with HGPS patients a premature ageing phenotype. MADA cells display an accumulation of prelamin A, an altered chromatin organization and dislocation of nuclear envelope-associated proteins [12,14].

Lamin-related functions and pathologies are largely dependent upon lamin interplay with multiple interacting partners. For example, the fact that the Ig-fold motif at the lamin C-terminus binds proliferating cell nuclear antigen (PCNA) suggests a mechanistic path for lamin implication in DNA replication [15]. Similarly, the lamin A/C interaction with 53BP1 to promote the DNA damage response (DDR) accounts for the DNA repair defects observed in lamin mutants [16]. The chromatin defects observed in HGPS patient cells probably reflect the interaction of lamins with the NURD chromatin remodelling complex [17]. Loss of another lamin-interacting protein, the Suv39h1 methyltransferase, has been shown to improve DNA repair and extend the lifespan of a progeroid mouse model with impaired prelamin A maturation, suggesting that HGPS causes Suv39h1-mediated epigenetic alterations in the chromatin [18]. Finally, lamin A/C interaction with the shelterin component TRF2 and the association of the lamina-associated polypeptide-α (LAP2α) with telomeres provide evidence for a role of lamin A and progerin in telomere homeostasis [6]. Although the biological significance of many of the lamins' interactions is not precisely defined, there is no doubt that further studies on these interactions and the discovery of new lamin-interacting partners will be instrumental to understand the mechanistic bases of the role of lamin in physiological and pathological processes [19].

Here, we provide a functional characterization of an additional lamin-interacting protein, AKTIP. AKTIP belongs to the ubiquitin E2 variant (UEV) enzyme subfamily, which comprises proteins that share sequence similarity with E2s but lack the conserved cysteine residue that is critical for the canonical E2 catalytic activity [20]. UEV functions are still ill defined; it has been suggested that they can act in combination with E2 conjugating enzymes in ubiquitylation processes [21,22]. We have recently shown that AKTIP and its *Drosophila* orthologue Pendolino (Peo) play roles at telomeres [23,24]. AKTIP directly binds the shelterin proteins TRF1 and TRF2 and interacts with the PCNA and RPA70 DNA replication factors, and its depletion results in general impairment of DNA synthesis and defective telomere replication. Collectively, our results indicate that AKTIP works in concert with TRF1 to ensure proper telomere replication.

The finding that both AKTIP and lamins interact with telomeres and with PCNA [3,6,23,25], along with the enrichment of AKTIP at the nuclear rim [23], prompted us to investigate whether AKTIP interacts with lamins. Here, we show that AKTIP co-purifies with A- and B-type lamins and partially co-localizes with lamins in interphase nuclei. In addition, we show that AKTIP depletion lowers lamin A expression and induces senescence hallmarks in human primary fibroblasts (HPFs).

## 2. Material and methods

### 2.1. Cells, vectors and virus

Human foreskin primary fibroblasts from healthy donors (provided by A. Orecchia, IDI, Rome, Italy) were used at early passages (from p5 to p10), unless otherwise specified. HeLa (ATCC CCL-2), 293T (ATCC CRL-11268), BJ-hELT cells and HPFs were cultured in DMEM (Invitrogen) supplemented with 10% FBS (Invitrogen). pCMV6-entry-AKTIP-MYC-FLAG (Origene), pTR-UF5 (provided by N. Muzyczka, University of Florida), or HDAC1-FLAG (Addgene, #13820) vectors were transfected in 293T cells according to Piersanti *et al.* [26]; cell extracts were analysed 72 h post-transfection, unless otherwise indicated. Second-generation recombinant lentiviruses (LVs) were produced and titrated for p24 antigen content and used for infections as previously described [26]. The LV-shAKTIP, LV-shTRF2 and LV-scramble (control, ctr) vectors were described previously [23]; the transfer vector pCDHblast MCSNard OST-LMNAΔ50 [17] (Addgene #22662) was used to produce LV-progerin. The multiplicity of infection (moi) used for all experiments was 3pg p24/cell on HPFs and 5pg p24/cell on HeLa cells. Transductions were performed in complete medium supplemented with 8 µg ml^−1^ polybrene (Sigma). After viral addition, cells were centrifuged for 30 min at 1800 r.p.m. at RT, incubated for 3 h at 37°C, and then transferred to fresh complete medium. Seventy-two hours post-infection, cells transduced with LVs obtained with pLKO.1-derived transfer vectors were subjected to selection in complete medium supplemented with 2 µg ml^−1^ puromycin (Sigma) and kept under these conditions for further analyses. Seventy-two hours post-transduction, cells transduced with LV-progerin were subjected to selection in complete medium supplemented with 10 µg ml^−1^ blasticidin (Sigma) and kept under these conditions for further analyses.

HeLa cells were synchronized at the G1/S boundary by a double-thymidine block. Cells were treated with 2 mM thymidine for 14 h, released in fresh medium for 10 h and treated again with 2 mM thymidine for 14 h. After double-thymidine treatment, cells were released in fresh medium and collected at the indicated time points.

MADA skin fibroblasts were obtained from a 12-year-old patient carrying the Arg527His LMNA homozygous mutation. Cell cultures were established and grown in DMEM-F12 (Gibco, Life Technologies) supplemented with 10% fetal bovine serum (Gibco, Life Technologies), 1% penicillin/streptomycin and 1% l-glutamine. The experiments were performed on cells at p12. HGPS fibroblasts were obtained from a patient carrying the G608G LMNA mutation [27] and were maintained in culture in DMEM supplemented with 10% fetal calf serum (Gibco) and 1% penicillin/streptomycin. Skin biopsies were obtained from patients and donors according to local and EU ethical rules following informed consent.

### 2.2. Western blotting

Whole protein extracts were obtained by treating cell pellets with lysis buffer (20 mM HEPES pH 7.5, 150 mM NaCl, 5 mM MgCl_2_, 0.5 mM EGTA, 0.25% NP-40, 1 mM DTT, 0.5 mM PMSF, 0.5 mM Na_3_VO_4_ and protease inhibitor cocktail, Roche). Samples were loaded onto pre-cast 4–12% gradient acrylamide gels (NuPage, Invitrogen). After electro-blotting, filters were incubated for 1 h with mouse monoclonal anti-AKTIP (1 : 400, Sigma), goat anti-actin-HRP conjugated (1 : 3500, Santa Cruz), mouse anti-lamin A/C (1 : 200, Santa Cruz), goat anti-lamin B1 (1 : 200, Santa Cruz), mouse anti-FLAG-HRP (1 : 1000; Sigma), rabbit anti-matrin 3 (1 : 1000, Santa Cruz), goat anti-importin 7 (1 : 200, Santa Cruz), mouse anti-trimethyl H3K9me3 (1 : 350, Millipore) and rabbit anti-TRF2 (1 : 3500, Novus Biologicals). Filters were then incubated with appropriate HRP-conjugated secondary antibodies (Santa Cruz; diluted according to the manufacturer's instructions), which were detected using the enhanced chemiluminescence system (ECL Plus, Amersham). Bands were imaged with the ChemiDoc MP imager (Bio-Rad) and band intensities were quantified using the Image Lab software (Bio-Rad).

### 2.3. Mass spectrometry

Seventy-two hours after transfection with the AKTIP-FLAG, pTR-UF5 or HDAC1-FLAG (Addgene, #13820) vectors in 293T cells, extracts were obtained by lysing the cells with 20 mM Hepes pH 7.5, 150 mM NaCl, 5 mM MgCl_2_, 0.5 mM EGTA, 0.25% NP-40, 1 mM DTT, 0.5 mM PMSF, 0.5 mM Na_3_VO_4_ in the presence of a protease inhibitor cocktail (Roche). Immunoprecipitation was performed by incubation of 2 mg of protein extract with 5 µg of anti-FLAG M2 antibody (Sigma) or 5 µg of mouse IgG (Sigma), both conjugated to G-sepharose four fast flow beads (GE Healthcare). Precipitated complexes were eluted using acid glycine (pH 2.5) and 2% Triton X-100 (Sigma) and then resuspended in SDS/PAGE loading buffer (Invitrogen). For mass spectrometry (MS), samples were separated onto pre-cast 4–12% gradient acrylamide gels (NuPage, Invitrogen). Entire gel lanes were cut into 10 similar slices, subjected to in-gel tryptic digestion, and used for LC-MS/MS analysis. Raw spectra were analysed by Bioworks Browser 3.3.1. When proteins were present both in anti-FLAG and in anti-IgG antibody samples, they were selected by applying the emPAI ratio [28] equal or higher than 1.5. The UNIPROT IDs of putative AKTIP interactors were uploaded in the Protein Knowledgebase (UniProtKB) and categorized based on Gene Ontology (GO) annotation terms by using Cytoscape v 2.8.3 (see the electronic supplementary material for full MS methods).

### 2.4. GST pulldown

AKTIP-GST and AKTIP-GST truncations were previously described [23]. Protein purification and GST pulldown from 293T cell extracts were carried out as previously described [23]. Immunoblotting was performed as described above.

### 2.5. Immunostaining

If not otherwise specified, for immunostaining of interphase nuclei samples were pre-permeabilized as previously described [23], fixed with 3.7% formaldehyde for 10 min at 4°C, and permeabilized with 0.25% Triton X-100 in PBS for 1 min. Mitotic cells were fixed in the same way omitting the pre-permeabilization step. Samples were then blocked with 3% BSA and incubated for 1 h with the following primary antibodies all diluted in 1% PBS–BSA: mouse anti-AKTIP (1 : 20, Sigma), mouse anti-FLAG-HRP (1 : 500, Sigma), goat anti-lamin B1 (1 : 50, Santa Cruz Biotechnology), goat anti-lamin A/C (1 : 100, Santa Cruz), rabbit anti-lamin A (1 : 200, Santa Cruz), mouse monoclonal α-tubulin-FITC conjugated (1 : 100, Sigma) and rabbit anti γ-tubulin (1 : 200, Sigma). Cells were then incubated for 45 min at RT with the following secondary antibodies diluted in PBS: FITC-conjugated anti-mouse (1 : 30, Jackson Immunoresearch), Alexa 555 anti-rabbit (1 : 200, Invitrogen), Alexa 568-anti-mouse (1 : 200, Invitrogen) or rhodamine-conjugated anti-mouse (1 : 30, Jackson Immunoresearch) or rhodamine-conjugated anti-goat (1 : 30, Santa Cruz) or FITC-conjugated anti-goat (1 : 50, Jackson Immunoresearch). After air drying, cells were mounted with DAPI-Vectashield (Vector Laboratories). All images were captured using a CoolSnap HQ CCD camera (Photometrics; Tucson, AZ, USA) connected to a Zeiss Axioplan fluorescence microscope equipped with an HBO 100 W mercury lamp.

SA-β-gal staining was carried out according to Dimri *et al*. [29]. Cells at different days post-infection were plated on coverslips for 24 h and then stained using the Senescent cell Histochemical Staining Kit (Sigma).

For prelamin immunostaining, distorted nuclei observation and chromatin analysis, cells were fifixed in 100% methanol at −20°C for 7 min or in 4% paraformaldehyde at RT for 10 min. Preparations were then incubated with goat anti-prelamin A (1 : 100, C-20, Santa Cruz) and rabbit anti-trimethyl H3K9 (1 : 100, Millipore) both diluted in PBS. Primary antibodies were detected by 1 h incubation with Alexa Fluor 568-conjugated anti-goat (1 : 800 in PBS, Invitrogen) and Alexa Fluor 488-conjugated anti-rabbit (1 : 500 in PBS, Invitrogen).

### 2.6. Flow cytometry

5-bromo-deoxyuridine (BrdU) at a final concentration of 45 µM was added to the culture medium 30 min before harvesting the cells. Cells were fixed in 70% ethanol, washed twice in PBS + 0.5% Tween 20, and incubated in 3 M HCl for 45 min. Cells were then exposed to anti-BrdU monoclonal antibody (Dako), to the secondary Alexa-Fluor488-conjugated antibody (Jackson) and counterstained with propidium iodide (PI, Sigma) in the presence of 1 mg ml^−1^ DNAse-free RNAse (Sigma) for 30 min at room temperature. Acquisition was carried out using a Beckman-Coulter Epics XL flow-cytometer and recorded data were analysed with the WinMDI software (developed by Joe Trotter; free download at http://en.bio-soft.net/other/WinMDI.html).

### 2.7. mRNA quantification

One-week post-transduction, cells were lysed by addition of TRIzol reagent (Invitrogen) and RNA extracted according to the manufacturer's instructions. After DNase treatment (Invitrogen), RNA was reverse transcribed into cDNA using an oligo d(T) primer and the Omniscript RT kit (Qiagen). To quantify target gene expression, specific primers (electronic supplementary material, table S1) were selected using Primer Express software (Applied Biosystems). Reactions were carried out as previously described [30].

## 3. Results

### 3.1. AKTIP binds nuclear envelope and nuclear matrix proteins

**Figure 1.**
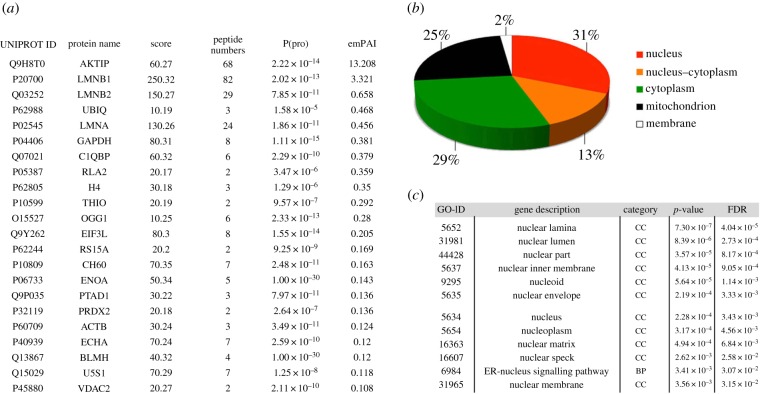
Identification of AKTIP-interacting proteins. (*a*) AKTIP-FLAG-interacting proteins immunoprecipitated from 293T whole-cell extracts with anti-FLAG antibody or IgG were subjected to MS. Raw data were analysed with the Bioworks Browser v,3.3.1 software. Number of peptides, score and protein probability are reported. Selected proteins are ordered by emPAI values, to a minimum of 0.1 (see the electronic supplementary material for a full list of interactors). (*b*) Subcellular locations of AKTIP interactors obtained using UniProtKB. (*c*) Functional enrichment of AKTIP interactors, evaluated using Cytoscape 2.8.3. CC, Cellular Components; BP, Biological Processes, FDR, false discovery rate.

As a first step towards the identification of AKTIP-interacting partners, we analysed by MS the proteins that co-purify with AKTIP. 293T cells were transfected with a vector encoding an AKTIP-FLAG fusion protein [23], or a control HDAC1-FLAG expression plasmid, or an empty pTR-UF5 vector. Previous work has shown that AKTIP-FLAG accumulates at the nuclear rim just as the untagged AKTIP protein [23] (see also electronic supplementary material, figure S1A). The AKTIP-FLAG fusion protein also displayed the expected molecular weight and was detected by both anti-FLAG and anti-AKTIP antibodies (electronic supplementary material, figure S1*b*). Seventy-two hours after transfection, we precipitated AKTIP-FLAG from whole-cell extracts using an anti-FLAG antibody; MS was carried out on immunoprecipitates following polyacrylamide gel electrophoresis (electronic supplementary material, figure S1B). To detect AKTIP bona fide interactors and eliminate false positives, we filtered raw data from three independent experiments using the Bioworks Browser 3.3.1 software (see Material and methods). These analyses led to the identification of 45 AKTIP-interacting proteins (figure 1*a* and electronic supplementary material, figure S2). Using UniProtKB, we found that these proteins localize in the nucleus (31%), in the nucleus and the cytoplasm (13%), in the cytoplasm (29%) or in the mitochondria (25%) (figure 1*b*). Cytoscape enrichment analysis further revealed that the nucleus and especially the nuclear lamina are GO categories significantly overrepresented among AKTIP-interacting partners (figure 1*c*). AKTIP-interacting proteins included lamins B1, B2 and A/C (figure 1*a*), importin 7, a nuclear-pore-associated β-family import receptor [31] and matrin 3, an internal nuclear matrix component that interacts with lamin A [32] (electronic supplementary material, figure S2).

**Figure 2.**
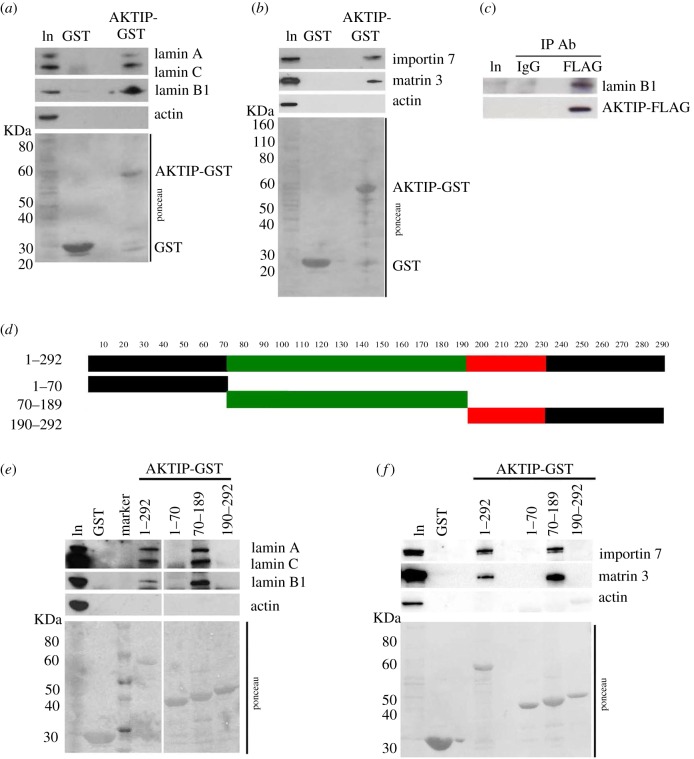
AKTIP interacts with lamins and with other nuclear proteins. (*a*,*b*) Purified AKTIP-GST or GST alone was used to pull down AKTIP-interacting proteins from 293T extracts. Western blotting shows that lamin A/C and lamin B1 (*a*) along with importin 7 and matrin 3 (*b*) interact with AKTIP-GST but not with GST alone. (*c*) Co-IP from extracts of 293T cells expressing AKTIP-FLAG showing that AKTIP and lamin B1 interact *in vivo*. (*d*) Schematic of AKTIP-GST truncations used in GST pulldown experiment from 293T cell extracts. These truncations include a disordered region at the N-terminus (black), the central region including the UEV portion (green) and a C-terminal region comprising 2 α-helices (red) and a disordered region (black). (*e*,*f*) Western blots showing that lamin A/C and lamin B1 (*e*) along with importin 7 and matrin 3 (*f*) interact with the central region of AKTIP.

To validate these MS data, we performed a pulldown assay using bacterially purified AKTIP-GST to capture proteins from 293T cell extracts. Consistent with the MS data, precipitates included lamins B1 and A/C, matrin 3 and importin 7. In addition, we found that an anti-FLAG antibody precipitates lamin B1 from AKTIP-FLAG-expressing 293T cells (figure 2*a–c*).

We next determined the AKTIP region(s) that interact with the nuclear envelope proteins. We used three AKTIP truncations fused with GST including the N-terminal disordered region (aa 1–70), the central region comprising the UEV portion (aa 70–189) and the C-terminus consisting of 2 α-helices and a distal disordered portion (aa 190–292) (figure 2*d*) [23]. GST pulldown showed that only the central region of AKTIP interacts with A- and B-type lamins, importin 7 and matrin 3 (figure 2*e,f*).

### 3.2. AKTIP partially co-localizes with lamins

**Figure 3.**
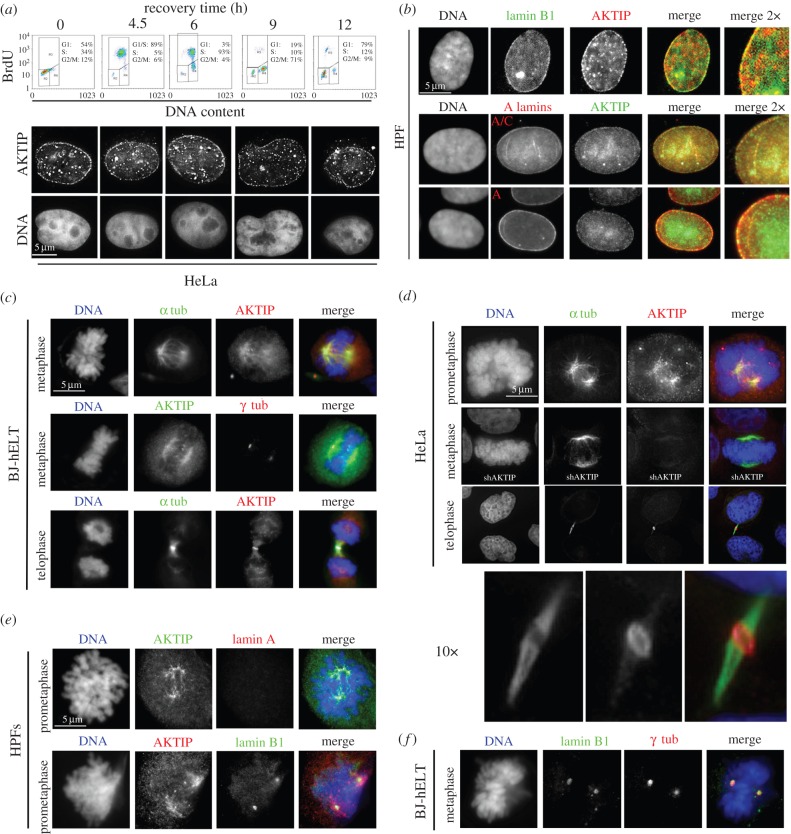
Partial co-localization of AKTIP and lamins in interphase and mitotic nuclei. (*a*) Interphase nuclei of HeLa cells at different recovery times after thymidine block immunostained for AKTIP display AKTIP enrichment at the nuclear rim and in intranuclear aggregates. (*b*) HPF nuclei stained for AKTIP and lamin B1, and AKTIP and either lamin A or lamin A/C. Note the partial colocalization of AKTIP with lamins B1 and A/C. (*c*) Mitotic divisions of BJ-hELT fibroblasts immunostained for AKTIP and either α- or γ-tubulin. Note that AKTIP is enriched in the spindle region, at the centrosomes and at the midbody. (*d*) Mitotic divisions of HeLa cells immunostained for AKTIP and α-tubulin. Note the AKTIP localization at the spindle poles and the midbody and the absence of AKTIP signals in AKTIP-depleted (shAKTIP) cells. (*e*) Dividing HPFs co-immunostained for both AKTIP and lamin A, and both AKTIP and lamin B1. Note the diffuse localization pattern of lamin A and the enrichment of both AKTIP and lamin B1 at the centrosomes. (*f*) Dividing BJ-hELT co-immunostained for lamin B1 and γ-tubulin. Note the co-localization pattern at the centrosomes. DNA was stained with DAPI.

Previous work has shown that A- and B-type lamins form two prominent nuclear meshworks with points of co-localization during interphase [33]. During mitosis, after dissolution of the nuclear lamina, A-type lamins exhibit a diffuse localization. By contrast, B-type lamins associate with the spindle matrix, where they are thought to provide a scaffold to promote microtubule assembly and lamina reorganization at the nuclear periphery at the end of mitosis [9,10]. Given the interaction between AKTIP and lamins, we investigated AKTIP localization. We immunostained with an anti-AKTIP antibody HeLa cells harvested at different times after release from synchronization at the G1–S boundary by a double-thymidine block. We found that at all time points tested AKTIP was enriched at the nuclear rim, suggesting that this localization pattern is dominant during the cell cycle (figure 3*a*).

We next investigated whether AKTIP co-localizes with A- and B-type lamins in interphase nuclei. The analysis of HPFs stained for both AKTIP and lamin B1 showed that the two proteins have multiple co-localization points, both at the nuclear rim and in the nucleoplasm (figure 3*b*). Double staining of HPFs for AKTIP and either lamin A or lamin A/C showed a partial co-localization of AKTIP with A-type lamins at the nuclear periphery (figure 3*b*). We also observed partial colocalization of AKTIP intranuclear signals with lamin A/C signals (figure 3*b*).

To determine AKTIP localization during mitosis, we used a fixative containing 0.25% Triton X-100. This fixative results in a high level of protein extraction allowing a clear visualization of AKTIP, but it does not fully preserve spindle microtubules. We analysed AKTIP localization in BJ-hELT (BJ fibroblasts expressing hTERT and SV40 Large T) and HeLa cells and HPFs (figure 3*c*–*e* and electronic supplementary material, figure S4). In all cell types, antibody staining detected specific AKTIP-enriched mitotic structures, which were absent in AKTIP-depleted cells (shAKTIP) (figure 3*d*; see the electronic supplementary material, figure S3 for analysis of RNAi efficiency). The AKTIP signal was associated with structures at the cell poles that overlapped the spindle microtubules and the centrosomes. In addition, AKTIP concentrated to the midbody, a transient structure that connects the two daughter cells at the end of cytokinesis. The midbody contains microtubules that overlap in a narrow region at the centre of the structure. This region often appears dark after tubulin immunostaining (dark zone), because it is tightly associated with proteins that block antibody binding to tubulin [34]. Remarkably, AKTIP precisely encircles the dark zone of the midbody (figure 3*d*), suggesting a possible role of AKTIP in cytokinesis.

To ask whether AKTIP co-localizes with lamins during mitosis, we co-immunostained HPFs for AKTIP and either lamin A or B. As expected from previous work [9], lamin A did not co-localize with the AKTIP-enriched structures and was instead diffuse (figure 3*e*). By contrast, lamin B1 accumulated at the centrosomes and partially co-localized with AKTIP, which was enriched at both the centrosomes and the spindle (figure 3*e,f*).

### 3.3. Functional relationships between lamins and AKTIP

**Figure 4.**
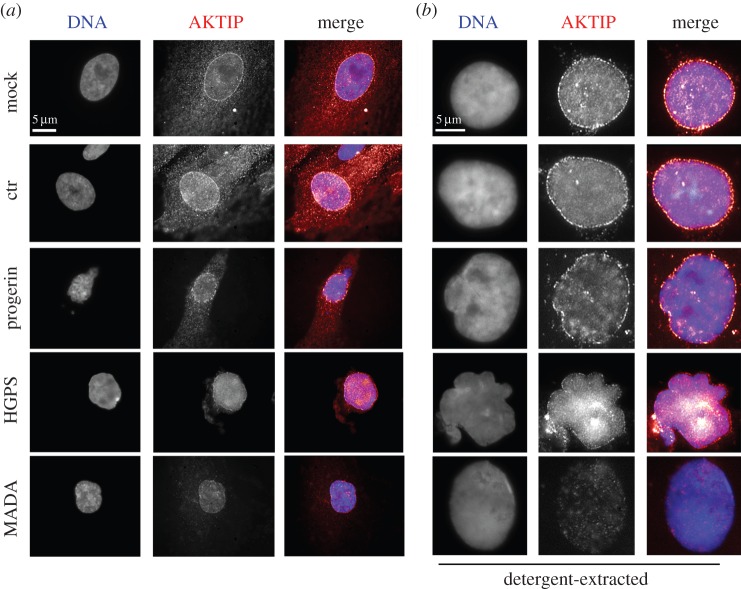
Abnormal AKTIP localization in lamin A mutant cells. Unextracted (*a*) or detergent-extracted (*b*) HGPS or MADA patient-derived fibroblasts, LV-progerin (progerin-expressing) HPFs and LV-control (ctr) or mock-treated HPFs stained for AKTIP and DNA (DAPI). Note that the AKTIP signals in the cytoplasm (*a*) and at the nuclear rim (*b*) of HGPS cells, MADA and progerin-expressing HPFs are weaker and more discontinuous than those observed in controls. In MADA nuclei, the AKTIP signal is almost undetectable.

Given the connection between AKTIP and lamins, we investigated the functional relationships between these proteins. We first asked whether mutations in lamin genes affect AKTIP localization. We analysed AKTIP localization in HPFs from patients affected by HGPS or MAD type A (MADA). The HGPS mutation exposes a cryptic splice site in the *LMNA* gene, leading to a lamin A form deleted of 50 aa and permanently farnesylated [35,36]. MADA cells, which carry the R527H *LMNA* mutation that replaces an arginine with a histidine at position 527, are characterized by accumulation of prelamin A and progeroid traits [37]. In addition, we examined AKTIP localization in HPFs transduced with a progerin-coding lentivector (LV-progerin; electronic supplementary material, figure S5). Immunostaining for AKTIP showed that in untreated control cells AKTIP is enriched in both the cytoplasm and the nucleus (particularly at the nuclear rim), while in detergent-extracted control cells AKTIP localization was restricted to the nucleus (figure 4*a,b*). In unextracted LV-progerin cells expressing both endogenous lamin A and progerin, and more effectively in unextracted HGPS cells, which are heterozygous for the *LMNA* mutation, the AKTIP signal in the cytoplasm was reduced and the nuclear rim staining was weaker and more discontinuous than in controls (figure 4*a*); the defective AKTIP localization at the nuclear rim of these cells was more evident in preparations extracted with Triton X-100 (figure 4*b*). In MADA cells, homozygous for the *LMNA* mutation, AKTIP was almost undetectable (figure 4*a,b*).

**Figure 5.**
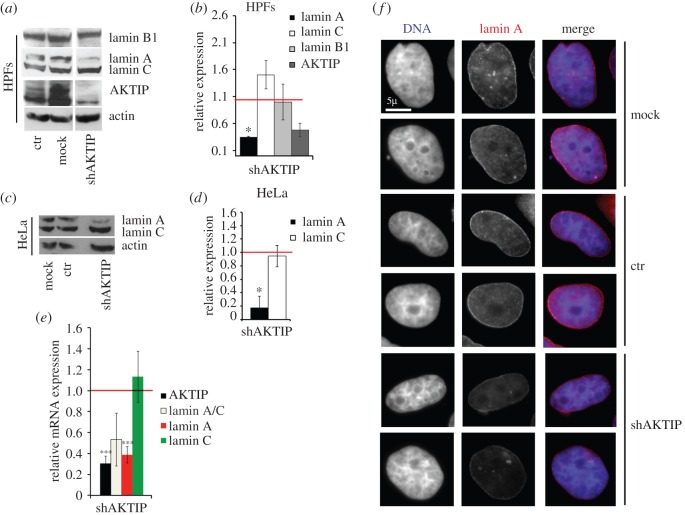
Functional relationships between AKTIP and lamins. (*a*,*c*) Western blotting performed at 7 days post-infection showing that in both HPFs (*a*) and HeLa cells (*c*) AKTIP depletion results in a decrease in lamin A without affecting the lamin C or B1 levels; the full blot of HPF extracts is shown in the electronic supplementary material, figure S3*c*. (*b*,*d*) Band intensity quantification (±s.d.) relative to control (arbitrarily set to 1, red horizontal line) from three independent experiments performed with HPFs (*b*) or HeLa cells (*d*); *p < 0.05 in Student's *t*-test. (*e*) Lamins and AKTIP mRNA levels determined by Q-PCR in HeLa cells 7 days after LV infection. The horizontal red line represents the control level arbitrarily set to 1. Lamin A mRNA level (±s.d.) is significantly reduced compared with control (***p < 0.001 in Student's *t*-test from three independent experiments), while lamin C mRNA is not significantly affected. Note that total LMNA RNA (including the A and C splicing products) shows an intermediate value compared with lamin A and C. (*f*) Mock-treated HeLa cells, or infected with either a control vector or with shAKTIP, stained with anti-lamin A. Note the weak lamin A signal in AKTIP-depleted nuclei.

We next investigated whether loss of AKTIP affects lamin expression. We reduced the AKTIP level by RNAi in early passage HPFs and HeLa cells (electronic supplementary material, figure S3). Western blotting performed on extracts from both cell types collected at 7 days post-infection showed that AKTIP depletion results in a substantial decrease in lamin A compared with control cells, without affecting lamin C and lamin B1 levels (figure 5*a*–*d*). In addition, Q-PCR-mediated mRNA quantification in HeLa cells at 7 days after shAKTIP infection showed a significant decrease in the lamin A mRNA level compared to control (figure 5*e*). However, AKTIP deficiency did not affect the level of lamin C mRNA, which is the main alternative splicing product of the *LMNA* gene. Consistent with these results, lamin A immunostaining in AKTIP-depleted HeLa cells showed a substantial reduction of the lamin A signal, which, however, maintained its normal association with the nuclear rim (figure 5*f*).

We have previously shown that AKTIP is required for telomere replication and that its depletion generates telomere-associated DNA repair foci [23]. Depletion of the TRF2 shelterin component, which physically interacts with lamin A/C [6], also causes telomere dysfunction and triggers the DDR [38]. We thus wondered whether lamin A reduction observed in AKTIP-depleted cells could be generated by dysfunctional telomeres. To test this possibility, we monitored lamin expression in shTRF2 HeLa cells. TRF2 depletion did not result in lamin A/C reduction, indicating that the effect of AKTIP depletion on lamin A is specific and not a general consequence of DDR triggered by dysfunctional telomeres (electronic supplementary material, figure S6).

### 3.4. AKTIP depletion results in cell senescence

**Figure 6.**
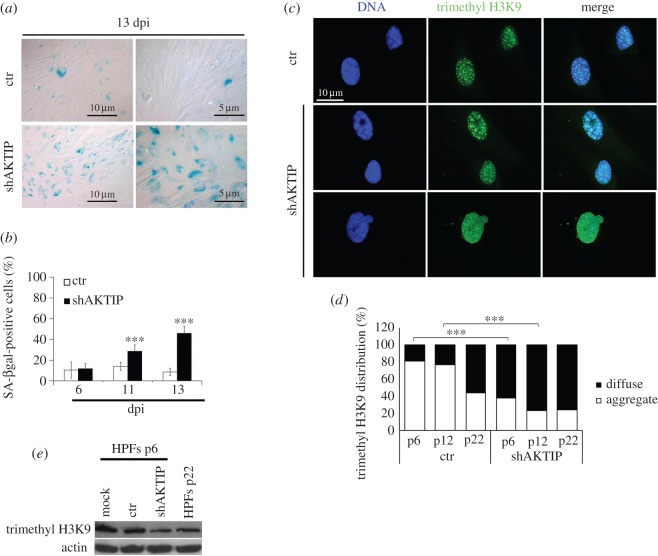
AKTIP depletion causes cell senescence and epigenetic alterations of chromatin. (*a*,*b*) AKTIP-depleted (shAKTIP) HPFs exhibit a significant increase of SA-βgal-positive cells compared with control at both 11 and 13 days post-infection (dpi) with interfering lentivector. Example of SA-βgal-stained cells (*a*), and frequencies (±s.d.) of SA-βgal-positive cells from two independent experiments (50 microscope fields were counted in each experiment); ***p < 0.001 in Student's *t*-test (*b*). (*c*,*d*) Staining with an anti-trimethyl H3K9 antibody and DAPI shows that AKTIP-depleted (shAKTIP) p6 HPFs exhibit a higher frequency of nuclei with a diffuse trimethyl H3K9 distribution than matched controls, which often display intranuclear trimethyl H3K9 aggregates (*c*). Frequencies (±s.d.) of nuclei with diffuse trimethyl H3K9 from two independent experiments (50 microscope fields were counted in each experiment); ***p < 0.001 in the *χ*^2^-test (*d*). (*e*) Western blotting showing that AKTIP-depleted early passage (p6) HPFs contain a lower amount of trimethyl H3K9 compared with both control (ctr) and naturally aged (p22) HPFs.

Lamin mutations cause defects in nuclear structure, variations in heterochromatic marks and cell senescence [3]. These findings prompted us to inquire whether AKTIP depletion generates the same phenotypic traits elicited by *LMNA* mutations. Cell labelling with the empirical senescence marker SA-βgal [29] revealed that early passage HPFs (p6) at 11 and 13 days after shAKTIP infection (electronic supplementary material, figure S3*a*) exhibit a significant increase in senescent cells compared with controls (figure 6*a*,*b*). We next analysed K9 trimethylation of histone H3 (trimethyl H3K9), an epigenetic marker of cell senescence that is reduced in prematurely ageing lamin-defective cells [39]. Early passage wild-type cells show a clustered intranuclear distribution of trimethyl H3K9, while senescent cells exhibit a more diffuse distribution of this trimethylated histone [14,37,39]. Early (p6) and mid-late (p12) passage AKTIP-depleted HPFs (electronic supplementary material, figure S3*a*) displayed higher frequencies of nuclei with diffuse trimethyl H3K9 distribution than passage-matched controls (figure 6*c*,*d*). In addition, Western blotting showed that in AKTIP-depleted early passage (p6) HPFs, the trimethyl H3K9 level was reduced with respect to controls and was comparable to that observed in naturally aged HPFs at p22 (figure 6*e*).

**Figure 7.**
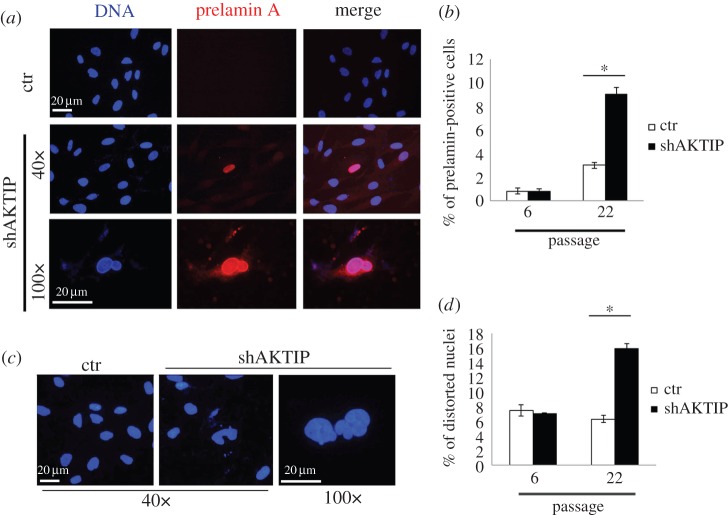
AKTIP depletion causes prelamin A accumulation and nuclear distortion. (*a*,*b*) AKTIP-depleted (shAKTIP) and control HPFs at different passages (p6 and 22) stained with the C20 anti-prelamin A antibody and DAPI (DNA) (*a*). Data from two independent experiments (50 microscope fields were counted in each experiment) show that AKTIP-depleted p22 HPFs display a higher frequency (±s.d.) of prelamin A-positive cells compared with passage-matched controls (ctr); *p < 0.05 in *χ*^2^-test. (*b*). (*c*,*d*) AKTIP-depleted HPFs at p22 exhibit frequent nuclei with distorted morphology (*c*). Data from two independent experiments (50 microscope fields were counted in each experiment) show that frequency (±s.d.) of distorted nuclei in AKTIP-depleted HPFs is significantly higher than in matched controls; *p < 0.05 in *χ*^2^-test (*d*).

Previous studies on naturally aged cells have shown that they accumulate unprocessed lamin A (prelamin A) [40]. This accumulation exacerbates the disease traits in HGPS and MADA patients, and eventually leads to nuclear deformation [12,14]. To ask whether AKTIP depletion results in a similar phenotype, we treated HPFs at different passages (p6 and p22) with shAKTIP (electronic supplementary material, figure S3*a*) and examined 7 days post-infection cells immunostained with the C20 antibody, which specifically reacts with prelamin A. shAKTIP HPFs at p22 displayed a higher frequency of prelamin A-positive cells than passage-matched control HPFs (figure 7*a*,*b*). We also examined AKTIP-deficient HPFs for distorted nuclei, which have been described in lamin mutant cells [12,14,39]. We found that AKTIP-depleted HPFs at p22 exhibit a significantly higher frequency of distorted nuclei than control cells (figure 7*c*,*d*).

Taken together, these data indicate that AKTIP depletion results in phenotypic traits that are characteristically associated with lamin A mutations.

## 4. Discussion

We have recently shown that AKTIP interacts with the PCNA and RPA70 components of the DNA replication machinery and with the shelterin subunits TRF1 and TRF2. Consistent with these results, we found that AKTIP is required for both general DNA replication and telomere replication [23]. Our MS experiments have shown that AKTIP also interacts with A- and B-type lamins, matrin 3, importin 7 and several mitochondrial proteins. The AKTIP interaction with lamins, matrin 3 and importin 7 was further validated by GST pulldown.

We focused on the relationships between AKTIP and lamins and found that these proteins exhibit similar localization patterns in interphase nuclei, with AKTIP enriched at the nuclear rim, showing partial co-localization with both the A- and B-lamin meshworks. We also found that AKTIP is enriched in structures that partially co-localize with the centrosomes and the spindle of mitotic cells. In addition, AKTIP forms a ring around the midbody during late cytokinesis. Previous studies in different cell types have shown that lamin B is also enriched at the centrosomes and spindle poles [10,41,42] as well as at the midbody [43]. It has been suggested the lamin network is part of the spindle matrix, a membranous network that favours spindle morphogenesis [10,42]. Thus, it appears that AKTIP and lamins are enriched in the same cellular compartments, a finding that suggests a functional interaction among these proteins.

Indeed, we found that AKTIP knockdown results in a specific reduction of lamin A at both the mRNA and the protein level. A specific downregulation of A-type lamins has been also found in other systems. For example, in mouse brains both lamin A and progerin expression is downregulated by miR-9, a brain-specific microRNA; miR-9 targets prelamin A 3′-UTR but not the lamin C transcript [44]. Lamin A and prelamin A downregulation and autophagic degradation have been also detected in response to retinoic acid and rapamycin treatments, in the absence of concomitant lamin C variations [27,45]. Our current results do not allow us to envisage a precise model to explain how AKTIP-deficient cells specifically downregulate lamin A expression. It is possible that loss of AKTIP lowers the production of lamin A by affecting the splicing of *LMNA* RNA or by controlling both prelamin A degradation and *LMNA* gene expression [46]. It is also conceivable that the prelamin A mRNA is downregulated by a microRNA. However, this hypothetical microRNA cannot be miR-9, because it was not found in HeLa cells [44]. The most intriguing aspect of the AKTIP-mediated regulation of prelamin A mRNA is that AKTIP interacts physically with both lamin A/C and lamin B. This finding raises the enthralling possibility that AKTIP regulates prelamin A mRNA expression through an interaction with one or more lamin types.

We also found a modest increase (from 3 to 9%) in the frequency of prelamin A-positive cells in late passage samples of AKTIP-depleted cells. This finding is in apparent contrast with the downregulation of lamin A observed in in early passage AKTIP-depleted cells. However, it should be considered that prelamin A accumulation reflects a slowdown in prelamin A processing, a phenomenon that characterizes the senescent phenotype associated with physiological and premature ageing [47]. It is thus possible that the accelerated senescence of AKTIP-depleted cells strongly affects prelamin A processing leading to an accumulation of this protein even in cells in which level of lamin A mRNA was initially reduced compared with controls.

We showed that AKTIP is mislocalized in LV-progerin and HGPS cells and in cells from MADA patients that accumulate prelamin A [12,14,39]. These findings raise the possibility of a role of AKTIP in progerin-induced pathogenesis. Another possible contributing factor to HGPS pathogenesis is the lamin A-interacting LAP2α protein [48,49]. It has been also shown that LAP2α transiently binds telomeres [50] and that progerin has the ability to reduce LAP2α association with telomeres [48]. Here, we have not addressed the relationships between AKTIP and LAP2α but we have shown that in HGPS cells the nuclear localization of AKTIP is strongly reduced compared with controls, suggesting a possible effect on proper telomere replication. These data, together with the cellular senescence observed in AKTIP-depleted cells, reinforce the idea of a functional interconnection between lamins and AKTIP and support the hypothesis of a possible involvement of AKTIP in progeroid syndromes.

Our MS analyses, in addition to lamins, identified matrin 3, importin 7 and several mitochondrial proteins as AKTIP partners. The functional relationships between AKTIP and these interacting partners deserve further investigation. In this respect, it should be noted that previous studies have shown that proteins involved in telomere maintenance such as the ATM kinase and the shelterin subunit TIN2 localize to mitochondria and regulate mitochondrial activity [14,51].

Altogether, our results identify AKTIP as a new lamin interactor, expanding the connection between telomeres and lamins and, more in general, between telomeres and nuclear envelope. In addition, the interplay between AKTIP and both lamin A and its aberrant forms responsible for HGPS and MADA suggests a possible involvement of AKTIP in the pathogenesis of these diseases.

## Supplementary Material

Supplementary material

## Data accessibility

The datasets supporting this article have been uploaded as part of the electronic supplementary material.
