# Correction to: ‘The telomeric protein AKTIP interacts with A- and B-type lamins and is involved in regulation of cellular senescence’ (2016), by Burla *et al.*

**DOI:** 10.1098/rsob.240314

**Published:** 2024-11-13

**Authors:** Romina Burla, Mariateresa Carcuro, Mattia La Torre, Federica Fratini, Marco Crescenzi, Maria Rosaria D'Apice, Paola Spitalieri, Grazia Daniela Raffa, Letizia Astrologo, Giovanna Lattanzi, Enrico Cundari, Domenico Raimondo, Annamaria Biroccio, Maurizio Gatti, Isabella Saggio

**Affiliations:** ^1^ Sapienza, Università di Roma, Dipartimento di Biologia e Biotechnologie, Roma, Italy; ^2^ Istituto Superiore di Sanità, Roma, Lazio, Italy; ^3^ University of Rome Torvergata, Roma RM, Italy; ^4^ Istituto di Genetica Molecolare, CNR Bologna, Pavia 40136, Italy; ^5^ Istituto di Biologia e Patologia Molecolari del CNR, Rome 00185, Italy; ^6^ Università degli Studi di Roma La Sapienza Facolta di Medicina e Psicologia, Roma, Lazio, Italy; ^7^ Istituti Fisioterapici Ospedalieri, Roma, Lazio, Italy

**Keywords:** lamin, telomeres, laminopathies, progeria, correction

Regarding the similarities detected by PubPeer between Fig. 4D from the PLOS Genetics publication (Burla *et al*., 2015) [1] and figure 2*e* from the Open Biology publication (Burla *et al*., 2016) [2], we wish to clarify the following.

The lower panels of Fig. 4D from PLOS Genetics 2015 (doi:10.1371/journal.pgen.1005167) and figure 2*e* from Open Biology 2016 (doi: 10.1098/rsob.160103) show the same Ponceau-stained filter, but the upper panels of the figures are very different, as they show staining of this filter with different antibodies (anti-TRF2 in PLOS Genetics and anti-lamins A, C and B1 in Open Biology). In the Open Biology paper, we reused the filter previously used in the PLOS Genetics paper. We stripped this filter with acidic glycine and re-probed it with anti-lamin antibodies. However, by mistake, we forgot to mention this fact.

We would also like to emphasize that the left part of figure 2*e* in Open Biology is redundant, as figure 2*a* of the same paper also shows that GST-tagged full-length AKTIP interacts with lamins A, C and B1.

In sum, the data fully support the interaction between the full-length AKTIP protein and lamins A, C and B1.
