# Supplementary material for: The telomeric protein AKTIP interacts with A- and B-type lamins and is involved in regulation of cellular senescence
Source: Open Biol. 2016 Aug 10;6(8):160103. doi: 10.1098/rsob.160103 (PMC5008010; doi:10.1098/rsob.160103)
Supplement: Supplementary material [file rsob160103supp1.docx]

**Supplementary material**

**Supplementary Methods**

**Mass spectrometry**

Gels were silver-stained (Silver Xpress Kit, Invitrogen). Entire lanes were cut into 10 similar slices, destained and subjected to in-gel tryptic digestion as previously described (Shevchenko et al. 1996). Supernatants from individual gel slices were used for LC-MS/MS analysis, performed using a nano-HPLC 3000 Ultimate (Dionex) connected in-line to an LTQ-XL linear ion trap (Thermo Fisher). Tryptic digests (20 µL) were loaded onto a homemade 9 cm x 75 µm-i.d. Silica PicoTip (8 ± 1 µm) column (PicoTip Emitter, NewObjective) packed with Magic C18AQ (5 μm particle size; 200 Å pore size, Michrom Bioresources Inc.) for chromatographic separations. Peptides were eluted along a 60 min linear gradient from 4% to 60% buffer B (95% acetonitrile, 0.1% formic acid) and electrosprayed directly into the mass spectrometer. The five most intense ions in the m/z 400-2000 mass range were sequentially selected and fragmented in CID mode. Dynamic exclusion of ions previously sequenced within 30 sec was applied. Raw spectra were analyzed by Bioworks Browser 3.3.1 using the Sequest 3.1 search engine against the Human SwissProt 2010 indexed database. Carboamidomethylation of cysteines and oxidation of methionine were specified as fixed and variable modifications, respectively. The minimal criteria for protein identification were: minimum of 2 distinct peptides; peptide probability < 10^-4^; peptide XCorr vs charge = 1.5 for charge +1; 2 for charge +2; 2.5 for charge +3; 3 for charge +4. When proteins were present in both the anti-FLAG and IgG (control) precipitates, they were selected by applying the emPAI ratio equal or higher than 1.5. The UNIPROT IDs of putative AKTIP interactors were uploaded in the Protein Knowledgebase (UniProtKB), functionally categorized based on GO annotation terms by using Cytoscape v. 2.8.3 and its plug-in Biological Networks GO tool program (BiNGO 2.44). Keratins and proteins with GO annotations referring to cell-junction or desmosome were considered non-specific contaminants (Hodge et al. 2013).

**Figure S1. Sample production for MS analysis.**

(A) Immunostaining with anti-FLAG antibody of 293T cells transfected with a vector encoding AKTIP-FLAG, showing that the tagged proteins is enriched at nuclear trim just as untagged AKTIP (see Figure 3). (B) Western blotting using both anti-FLAG and anti-AKTIP antibodies from extracts of 293T cells transfected with an AKTIP-FLAG coding vector, a control HDAC1-FLAG expressing-vector, or a control pTR-UF5 empty vector. (C) Seventy-two hours post-transfection 293T cell samples were immunoprecipitated with an anti-FLAG antibody or IgG and ran on polyacrylamide gels. Gels were Silver-stained.

**Figure S2. Identification of AKTIP-interacting proteins.**

Continuation of Figure 1A; see legend of figure 1.

**Figure S3. Levels of AKTIP expression after lentivirus-mediated RNA interference.**

(A, B) Expression of AKTIP mRNA relative to mock control in HPFs (A) and HeLa cells (B) transduced with LV-shAKTIP, with a control LV-scramble (ctr) or mock treated. mRNA was analyzed by Q-PCR seven days post infection. The columns represent the average value (± SD) from triplicate samples; the AKTIP mRNA level is relative to that of the mock sample at p12, which was arbitrarily set to 1 (* p < 0.05; ** p < 0.01; *** p < 0.001 in Student T test). (C) Original full picture of the western blot shown in figure 5A; the “el” lanes have been eliminated in figure 5A.

**Figure S4. Mitotic distribution of AKTIP in HPFs.**

HPF metaphase (top) and anaphase (bottom) immunostained for AKTIP; DNA was stained with DAPI. Note that AKTIP is enriched in the spindle region.

**Figure S5. Characterization of LV-progerin infected cells.**

Western blotting of extracts from LV-progerin-infected HPFs (7 days post-infection) and control (LV-ctr transduced cells and untreated mock) HPFs probed with an anti-lamin A/C antibody.

**Figure S6. TRF2 depletion does not affect lamin A.**

(A) HeLa treated with a TRF2 interfering lentivector (shTRF2, 7 days post infection) show a reduced TRF2 expression compared to mock (arbitrarily set to 1). The mRNA levels are the average of triplicate Q-PCR RNA measurements (± SD); a value of 1 arbitrarily was set to a single mock sample. * significantly different from controls in the Student’ t test with p < 0.05. (B, C) Western blotting of extracts from shTRF2 infected (7 days post infection) HeLa cells showing that TRF2 depletion affects neither laminA nor lamin C level (B). Lamin A band intensity quantification (± SD) relative to control from three independent Western blotting experiments (C). (D) TRF2-depleted cells and control HeLa cells (both at 7 days post infection) show similar levels of lamin A mRNA. The columns represent the average lamin A mRNA levels from triplicate measurements (± SD) by Q-PCR

**Table S1. Primers used for Q-PCR.**

| AKTIP Forward | TCCACGCTTGGTGTTCGAT |
| --- | --- |
| AKTIP Reverse | TCACCTGAGGTGGGATCAACT |
| LMNA Forward | ATGATCGCTTGGCGGTCTAC |
| LMNA Reverse | GCCCTGCGTTCTCCGTTT |
| lamin A Forward | CTCCTACCTCCTGGGCAACT |
| lamin A Reverse | AGGTCCCAGATTACATGATGCT |
| lamin C Forward | CTCAGTGACTGTGGTTGAGGA |
| lamin C Reverse | AGTGCAGGCTCGGCCTC |
| TRF2 Forward | TCCTCACGATGGCCAAAAAG |
| TRF2 Reverse | GCTGTTTATCTTCCTTCCCTGTACT |
| GAPDH Forward | TGGGCTACACTGAGCACCAG |
| GAPDH Reverse | GGGTGTCGCTGTTGAAGTCA |

**References**

Hodge, K, Have, ST, Hutton, L & Lamond, AI (2013) Cleaning up the masses: exclusion lists to reduce contamination with HPLC-MS/MS. *J Proteomics* 88:92-103. DOI: 10.1016/j.jprot.2013.02.023.

Shevchenko, A, Jensen, ON, Podtelejnikov, AV, Sagliocco, F, Wilm, M, Vorm, O, Mortensen, P, Shevchenko, A, Boucherie, H & Mann, M (1996) Linking genome and proteome by mass spectrometry: large-scale identification of yeast proteins from two dimensional gels. *Proc Natl Acad Sci U S A* 93:14440-5.

**Supplementary figure 1**


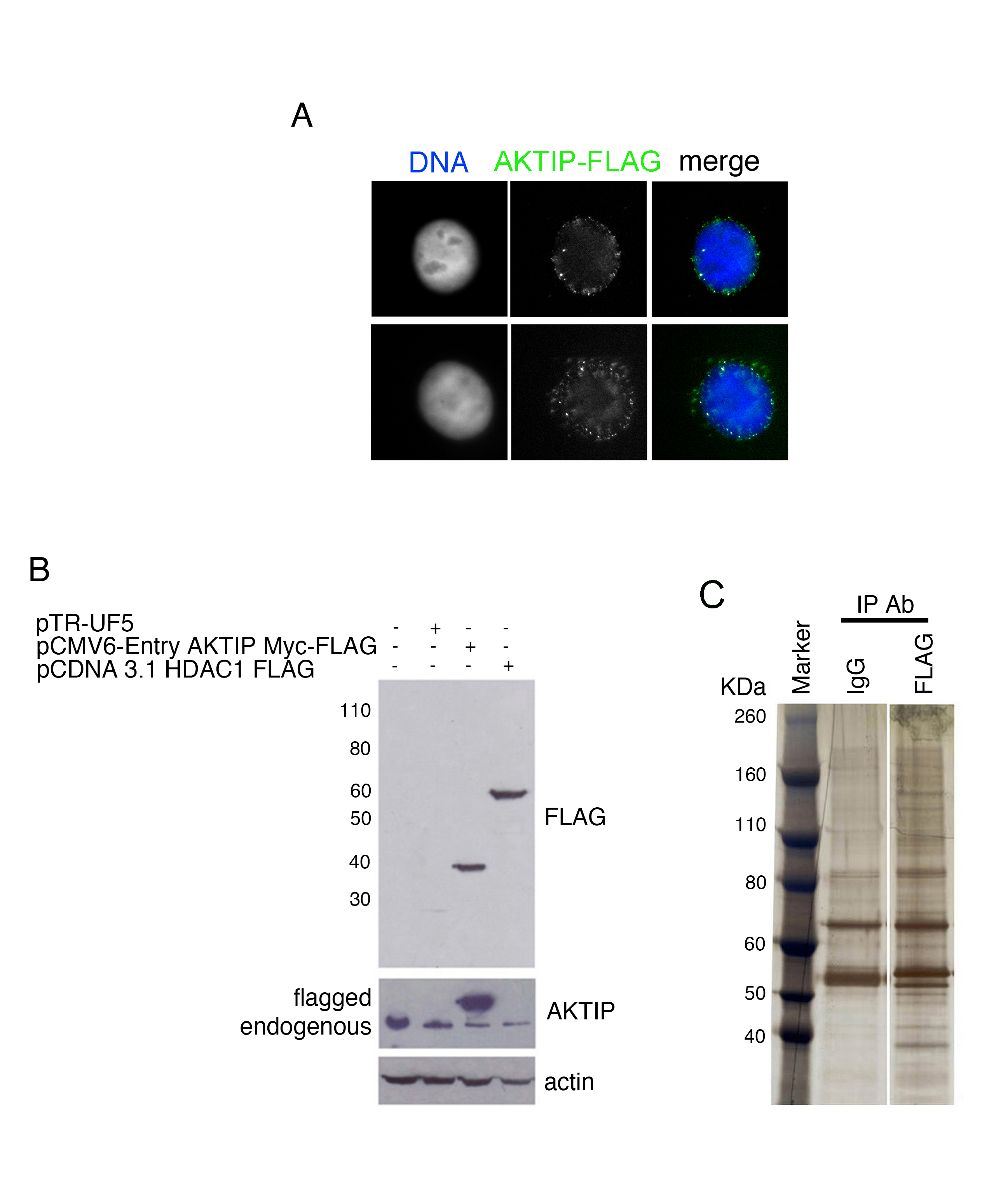


**Supplementary figure 2**

**
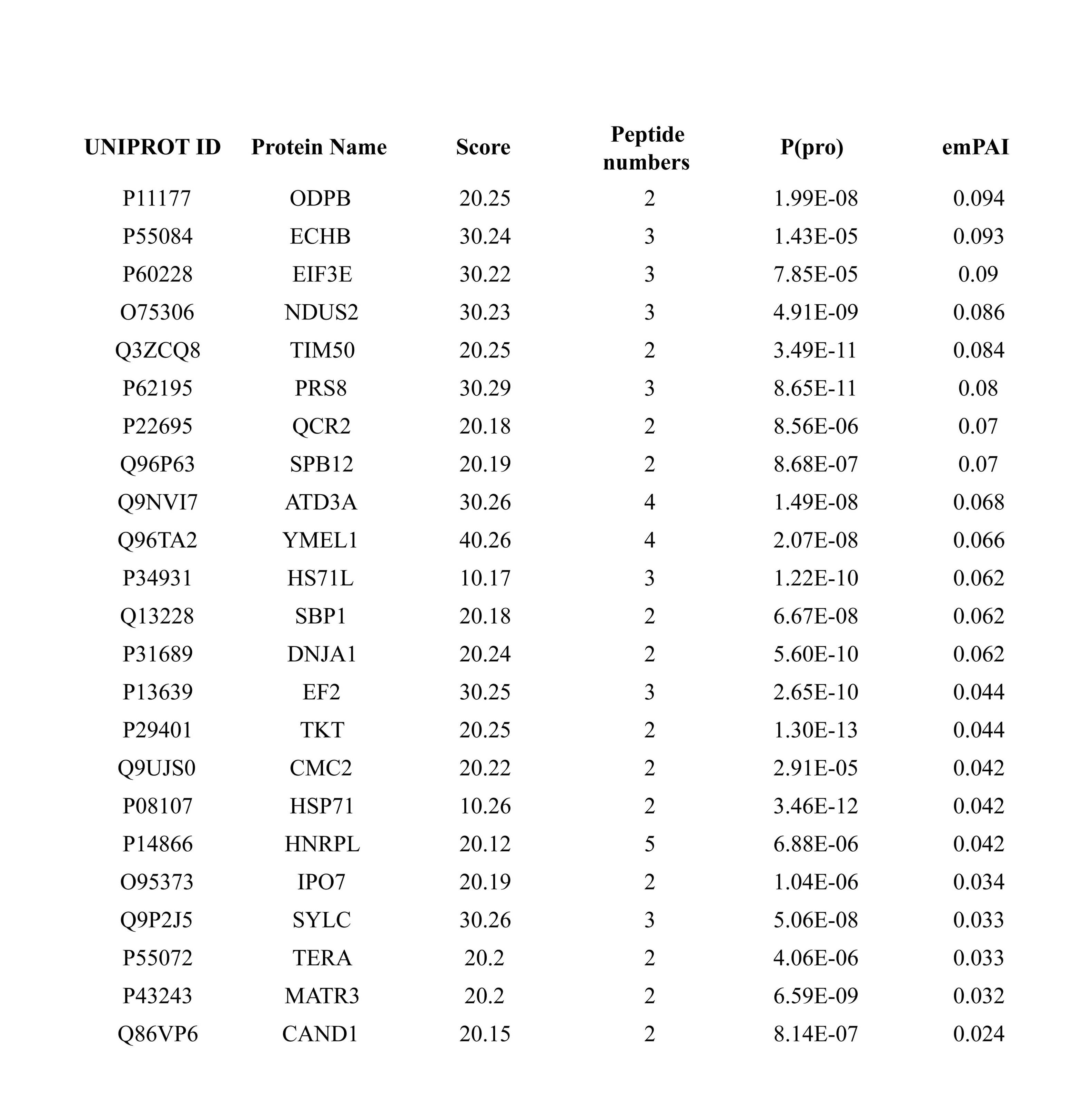
**

**Supplementary figure 3**

**
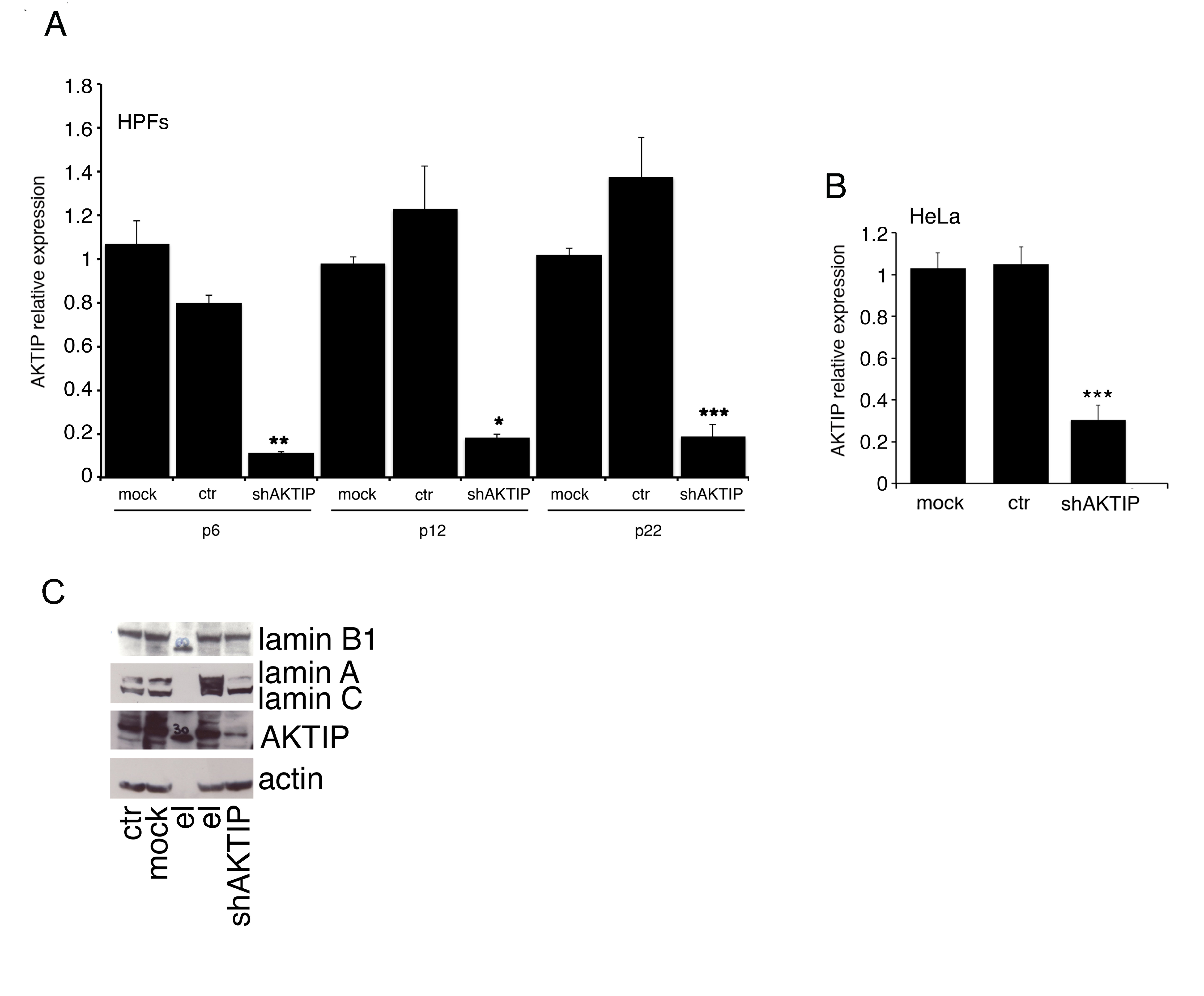
**

**Supplementary figure 4**

**
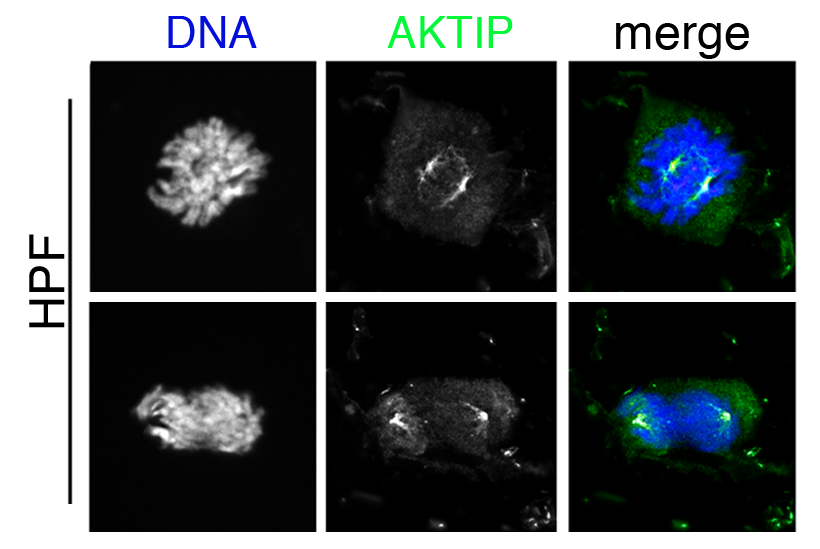
**

**Supplementary figure 5**

**
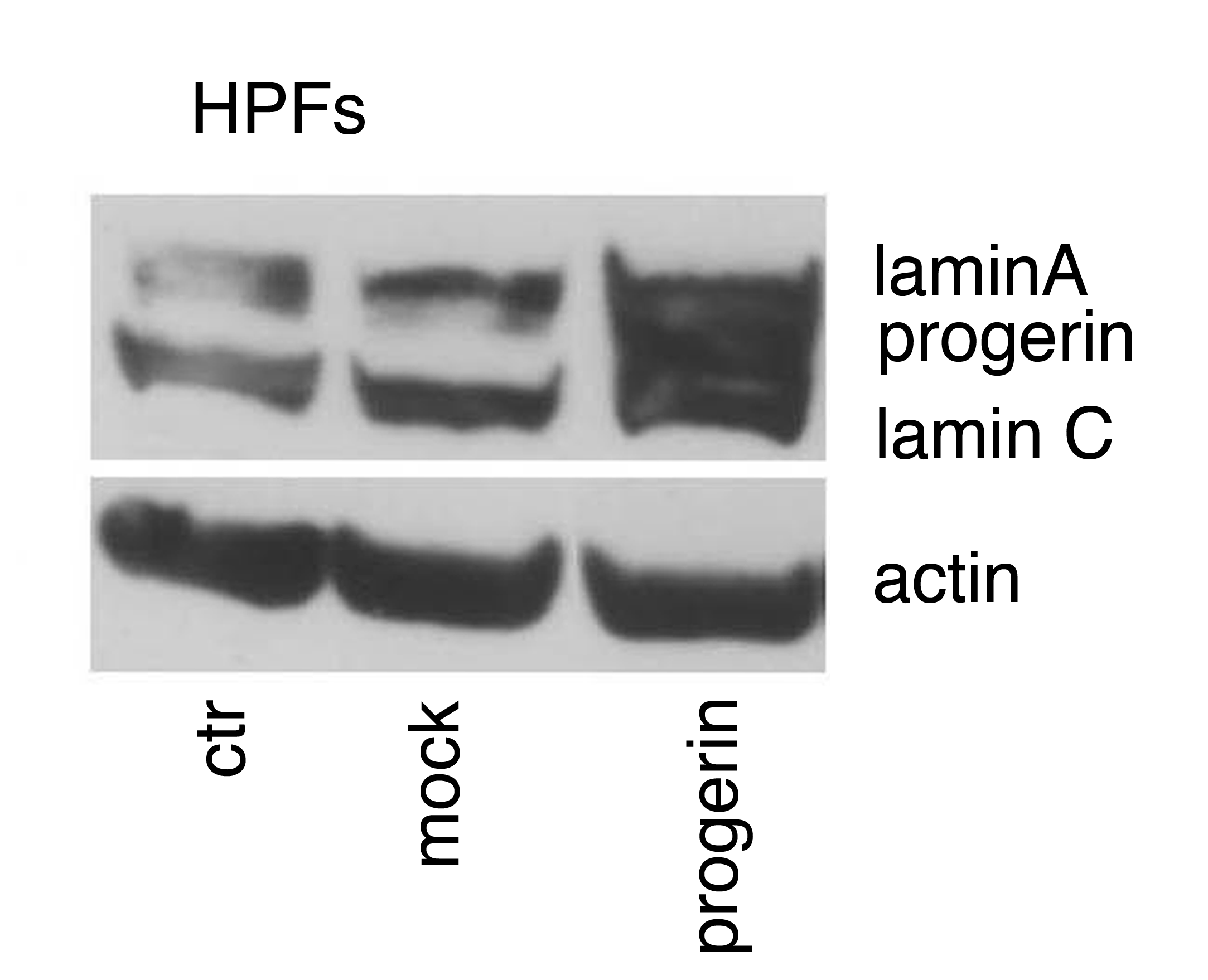
**

**Supplementary figure 6**

**
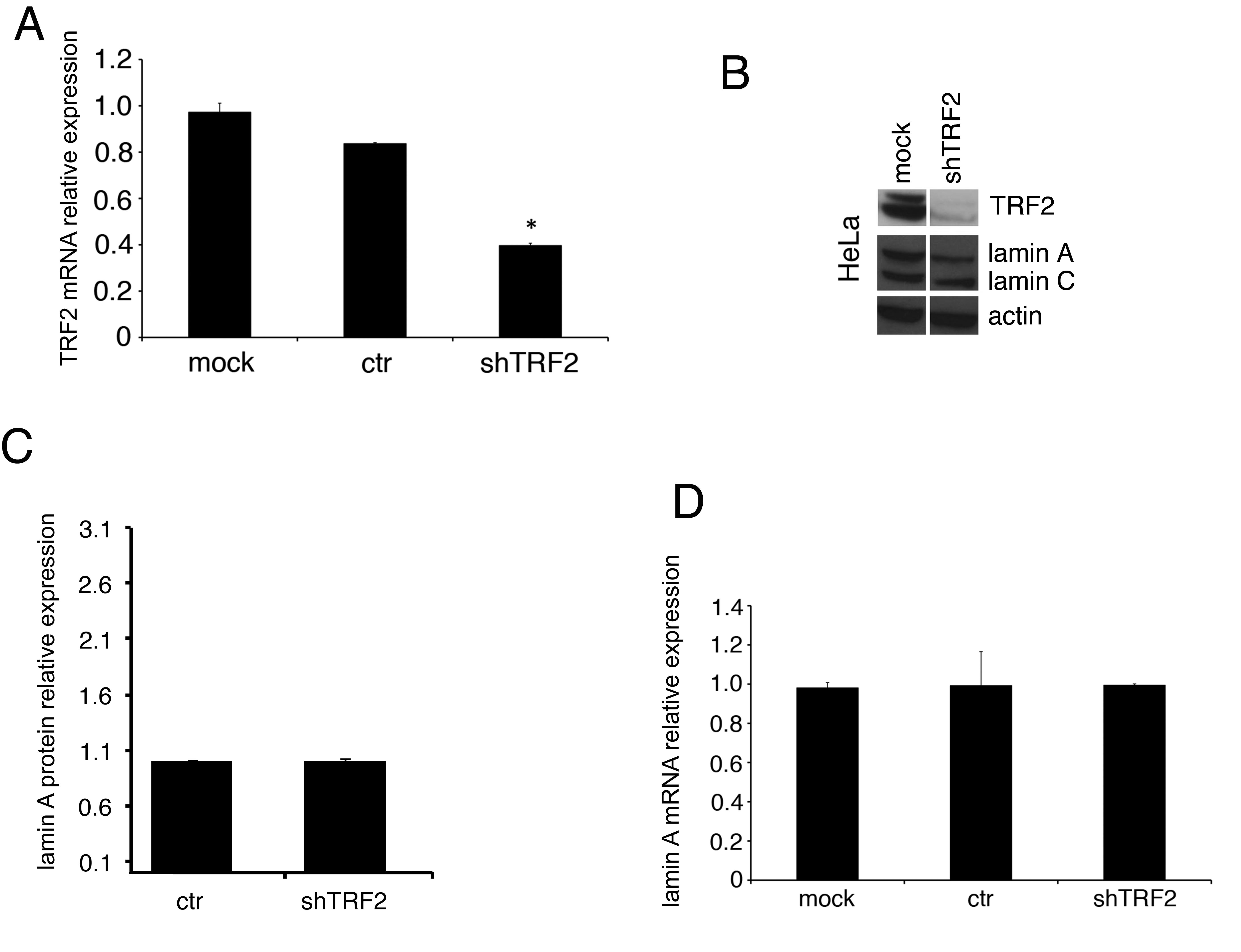
**
